# Oral administration of probiotic colony-like micro-nano system for immunoregulation of rheumatoid arthritis

**DOI:** 10.1016/j.apsb.2025.10.038

**Published:** 2025-10-31

**Authors:** Fangke Zhang, Tao Ding, Jiancheng Zheng, Nan Li, Zechuan Li, Xuefei Wang, Yawei Du, Weiguo Hu, Wenguo Cui, Weisheng Guo

**Affiliations:** a Department of Minimally Invasive Interventional Radiology, The Second Affiliated Hospital, School of Biomedical Engineering Guangzhou Medical University, Guangzhou 510260, China; b Department of Orthopaedics, Shanghai Key Laboratory for Prevention and Treatment of Bone and Joint Diseases, Shanghai Institute of Traumatology and Orthopaedics, Ruijin Hospital, Shanghai Jiao Tong University School of Medicine, Shanghai 200025, China; c Department of Geriatrics, Medical Center on Aging, Ruijin Hospital, Shanghai Jiao Tong University, School of Medicine, Shanghai 200025, China

**Keywords:** Micro-nano system, Nanomedicines, Melittin, Rheumatoid arthritis, Oral administration, Hydrogel microspheres, Autoimmune diseases, Peptide delivery

## Abstract

Rheumatoid arthritis (RA) is a chronic systemic autoimmune disease that requires long-term pharmacological management. Melittin, a peptide derived from bee venom, has shown promising therapeutic efficacy for RA by modulating immune balance. Given the critical role of the gut in immune regulation, oral administration of melittin could have significant clinical implications. However, this approach faces substantial challenges, including degradation by gastric fluids and off-target adverse effects, which compromise its efficacy and safety. To address these limitations, we developed an innovative orally administered, gut-targeted micro-nano system (SPM/AlgL) inspired by bacterial colonies. Herein, gas-shearing microfluidics is leveraged to monodisperse sialic acid-decorated peptide nanomedicines within calcium alginate microgels. These microspheres are then coated with probiotic biofilms, leveraging their acid resistance and intestinal adhesion properties. The biofilm coating effectively protects melittin from gastric degradation and enhances its accumulation in the mesenteric lymph nodes, thereby improving its targeting ability to inflammatory sites and reducing adverse effects. By modulating the Th1/Th2 and Th17/Treg ratios in the mesenteric lymph nodes and spleen tissues, this system successfully alleviates immune responses and efficiently mitigates the progression of arthritis. Overall, this oral therapeutic strategy demonstrates significant potential for advancing the immunotherapy of RA and other systemic autoimmune diseases.

## Graphical abstract

An oral system was developed which protected melittin from gastric degradation and enhanced its accumulation in mesenteric lymph nodes, treating rheumatoid arthritis) by enhancing the system's immunomodulatory capacity.

**Figure:**
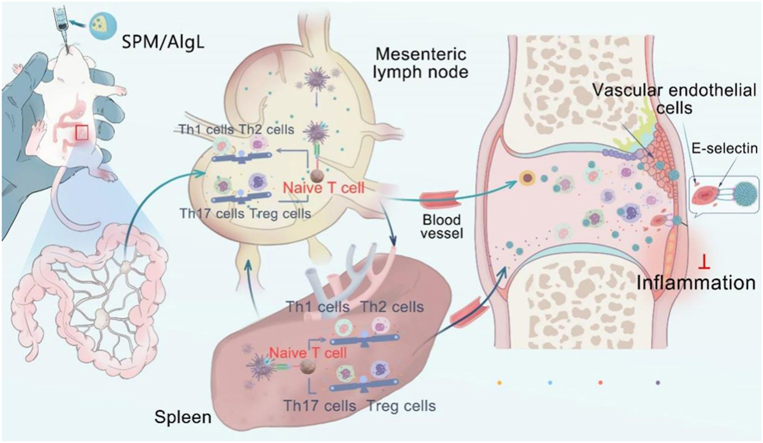

## 1 Introduction

**Scheme 1:**
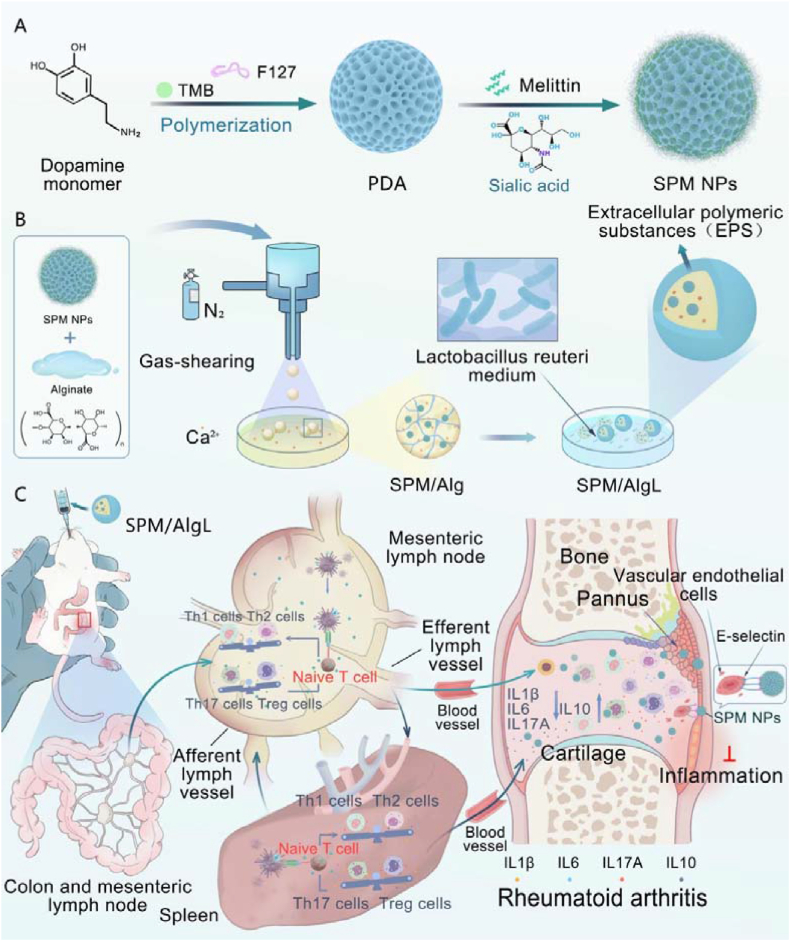
Bacterial colony-like micro-nano system for immunotherapy of rheumatoid arthritis. mesoporous polydopamine nanoparticles (PDA NPs) were synthesized *via* oxidative polymerization. (A) Melittin was encapsulated within these nanoparticles, and sialic acid was subsequently modified on their surface through an amide reaction to form SPM NPs. (B) SPM NPs were then loaded into calcium alginate microspheres, and the microspheres were coated with a probiotic biofilm to create the SPM/AlgL system. (C) Upon oral administration, SPM/AlgL targets the intestines, where it releases the nanoparticles (NPs). These NPs regulate the differentiation of T helper (Th) cells in the mesenteric lymph nodes, thereby restoring immune balance in the spleen. This mechanism ultimately facilitates immunotherapy for rheumatoid arthritis (RA).

Rheumatoid arthritis (RA) is a chronic, systemic autoimmune disease characterized by persistent inflammation, joint destruction, and significant pain. Current treatments for RA primarily focus on reducing inflammation and modulating the immune response. These include non-steroidal anti-inflammatory drugs (NSAIDs), corticosteroids, disease-modifying antirheumatic drugs (DMARDs), and biological agents^1^, ^2^. While these therapies can alleviate RA symptoms and have led to considerable advancements in treatment efficacy, the complex pathogenesis of RA makes it challenging to manage. As a result, patients often require lifelong therapy, which may lead to serious side effects due to long-term drug administration^3^, ^4^. The pathogenesis of RA involves intricate interactions among various immune cells, with a critical role played by the balance between different T helper (Th) cell subsets, including Th1/Th2 and Th17/Treg^5^, ^6^, ^7^, ^8^, ^9^, ^10^. In RA patients, the serum and synovial fluid exhibit an imbalance skewed toward Th1 and Th17 cells. This shift results in elevated levels of pro-inflammatory cytokines secreted by Th1 and Th17 cells, while anti-inflammatory cytokines produced by Th2 and Treg cells are reduced^11^. The dysregulation of Th cell subsets, particularly deficiencies or impaired function of Th2 and Treg cells, drives progressive joint and bone destruction, potentially leading to severe disability^12^, ^13^, ^14^. Therefore, therapeutic strategies aimed at restoring Th cell homeostasis are crucial for mitigating the effects of RA and improving patient prognosis^15^ (see Scheme 1).

Melittin, the main component of bee venom, has demonstrated significant potential as a therapeutic agent for RA by modulating Th cell responses and cytokine production, thereby regulating immune balance^16^. Several studies have explored nanodelivery strategies to target melittin directly to sites of joint inflammation *via* subcutaneous or intravenous administration^17^, ^18^. However, intravenous administration is impractical for long-term therapy, and drug accumulation in joints remains limited, often accompanied by severe adverse effects^19^, ^20^, ^21^. Since the intestine is the largest immune organ in the human body and RA is fundamentally driven by systemic immune imbalance^22^, ^23^, regulating intestinal immune homeostasis may offer an effective approach for treating RA. Therefore, we hypothesize that oral administration of melittin, targeting intestinal immune balance, could be a promising therapeutic strategy with significant clinical potential^24^. Melittin possesses several advantages as an oral peptide for RA treatment. First, its amphiphilic and cationic properties, stemming from its cell-penetrating nature, facilitate intestinal absorption. Second, melittin's rigid *α*-helical structure enhances its resistance to protease hydrolysis. Additionally, as a naturally derived peptide, melittin avoids the potential residues of chemical reagents, ensuring high oral safety. Despite these advantages, the clinical application of oral melittin faces several challenges. It is susceptible to degradation by gastric juices, with only a small fraction effectively absorbed into the bloodstream. Moreover, its high isoelectric point and strong non-selective cell penetration may lead to off-target effects and serious adverse reactions^25^, ^26^. Therefore, developing an effective oral delivery system is crucial to harnessing melittin's therapeutic potential for RA by modulating intestinal immune homeostasis.

Bacterial biofilms are dense and acid-resistant, which limits the penetration of small-molecule antibiotics and antimicrobial peptides. This characteristic poses a significant challenge in antimicrobial sterilization^27^, ^28^, ^29^. However, this very property has inspired the design of novel oral delivery systems. To address the aforementioned challenges, we have developed an innovative orally administered, gut-targeted micro-nano system, SPM/AlgL (SA-PDA@Melittin/Alg-*Lactobacillus reuteri* hydrogel microspheres), for intestinal immune modulation. This system leverages the acid resistance and intestinal adhesion properties of bacterial biofilms^30^.

In this study, melittin was first encapsulated within mesoporous polydopamine nanoparticles modified with sialic acids *via* electrostatic adsorption, and then loaded into calcium alginate microspheres encapsulated by a biofilm formed by *Lactobacillus reuteri*^31^, ^32^, ^33^, ^34^, ^35^. This dual-layer structure, comprising the biofilm and microspheres, protects the nanoparticles from degradation by gastric fluid and facilitates their smooth transport to the intestines^36^, ^37^, ^38^, ^39^, ^40^. Upon reaching the alkaline intestinal environment, the microspheres burst, releasing the nanoparticles, which then accumulate in the mesenteric lymph nodes. This accumulation promotes an increase in the ratio of Tregs to Th17 cells and Th2 to Th1 cells^41^. Furthermore, the nanomedicines circulating through the lymphatic system enhance the differentiation of T cells into Treg and Th2 cells in the spleen^42^, ^43^. This process is accompanied by a reduction in pro-inflammatory cytokines in the blood and inflamed joint cavities, thereby effectively restoring immune balance. This novel oral therapeutic strategy demonstrates significant potential for the immunotherapy of RA and other systemic autoimmune diseases.

## 2 Materials and methods

### 2.1 Materials

Anhydrous ethanol, trimethylbenzene, F127, tris, dopamine hydrochloride, sialic acid and sodium alginate (15–25 cP) were purchased from Sigma‒Aldrich Co., Ltd. (Shanghai, China). 1-(3-Dimethylaminopropyl)-3-ethylcarbodiimide (EDC), *N*-hydroxysuccinimide (NHS), and calcium chloride were purchased from Aladdin Biochemical Technology Co., Ltd. (Shanghai, China). Melittin was synthesized by Nanjing Taopu Biotechnology Co., Ltd. (Nanjing, China). *Lactobacillus reuteri* was purchased from Yiyan Biotechnology Co., Ltd. (Shanghai, China). Complete freund's adjuvant (CFA) was purchased from MedChemExpress (MCE). (Shanghai, China). Rat IL-17A ELISA Kit, Rat IL-10 ELISA Kit, Rat IL-6 ELISA Kit, and Rat IL-1*β* ELISA Kit were purchased from Thermo Fisher Scientific Co., Ltd. (Waltham, MA, USA). FITC Mouse Anti-Rat CD3, PE-Cy7 Mouse Anti-Rat CD4 (OX-35), Alexa Fluor 647 Mouse Anti-Rat IFN-*γ* (DB-1), PE Mouse Anti-Rat IL-4 (OX-81), Rat FoxP3 Alexa Fluor700 conjugated Antibody, IL-17A Monoclonal PerCP-Cyanine5.5 conjugated Antibody were purchased from Becton, Dickinson and Company (Franklin Lakes, NJ, USA). All chemicals were used without further purification.

### 2.2 Preparation and characterization of SPM NPs

A flask was sequentially charged with 100 mL of ultrapure water, 50 mL of anhydrous ethanol, 10 μL of trimethylbenzene, and 120 mg of F127. The mixture was stirred for 30 min to obtain a clear and transparent solution. Subsequently, 9 mL of Tris solution (10 mg/mL) and 70 mg of dopamine hydrochloride were added to the mixture and stirred for 48 h at room temperature. The resulting nanoparticles were collected by centrifugation and washed three times with anhydrous ethanol, then resuspended in anhydrous ethanol and stored at 4 °C.

For the surface modification, a solution containing 5 mg of sialic acid was prepared by dissolving it in 10 mL of filtered water. EDC and NHS were added to the solution in a molar ratio of 20:6.7, and the mixture was agitated for 2 h. Next, 10 mg of polydopamine (PDA) nanoparticles were introduced into the mixture and agitated for an additional 4 h. The modified nanoparticles (SP NPs) were collected by centrifugation. Finally, the 10 mg of SP NPs were evenly dispersed in deionized water and combined with melittin. The solution was then transferred to an ultrafiltration tube with a molecular weight cutoff of 50 kDa and centrifuged at 300 × *g* for 10 min. The concentration of melittin in the peptide nanomedicine was measured using High Performance Liquid Chromatography (HPLC). The detection of melittin was done by measuring its UV absorption at 220 nm. Eight concentrations of melittin ranging from 10 to 250 μg/mL, were chosen to measure the corresponding absorbance values. A standard curve of melittin was created. The absorbance of the melittin nanomedicine solution with a concentration of 1 mg/mL was measured. The melittin content was then calculated to determine the encapsulation rate and loading amount of the nanomedicine. The nanomedicine's encapsulation rate and drug loading capacity were ultimately computed. The nanomedicine's release in pH7.4 and pH5.6 media was simultaneously detected using HPLC, and the resulting release curves were generated.

### 2.3 Preparation and characterization of SPM/AlgL

Melittin nanomedicine was mixed with a 1% (*w*/*v*) sodium alginate solution at a ratio of 1:10 (nanomedicine to sodium alginate). This mixture served as the aqueous phase in a microfluidic system. The aqueous phase, containing sodium alginate and nanoparticles, was injected into another aqueous solution *via* coaxial needles. The shear force of a nitrogen gas flow (0.5 L/min) divided the aqueous phase into droplets with a diameter of approximately 200 μm. These droplets were collected in a calcium chloride (CaCl_2_, 0.2 mol/L) solution, forming calcium alginate microspheres, which were then stored at 4 °C. *Lactobacillus reuteri* was cultured in MRS medium at 37 °C for 24 h in an anaerobic incubator. The probiotic concentration was quantified as 1% using an ultraviolet spectrophotometer. A biofilm of *Lactobacillus reuteri* was developed on coverslips using the standard biofilm culture method. Coverslips were removed at specific time intervals, stained with DAPI, and examined using confocal laser scanning microscopy (CLSM) to optimize cultivation conditions. The modified microspheres were freeze-dried and evenly distributed into the *Lactobacillus reuteri* culture medium. The biofilm-coated microsphere culture solution was then strained through a 100 μm cell sieve to remove the culture medium and excess probiotic. The resulting biofilm-coated micro-nano-peptide drug system was dispersed in an MRS medium and stored at 4 °C. The morphology of a 100 μL solution containing the biofilm-coated micro-nano peptide drug was examined using scanning electron microscopy (SEM). Energy-dispersive spectroscopy (EDS) was employed to analyze the elemental distribution in both the microspheres and biofilm-coated microspheres. Additionally, the formation and size of biofilms were assessed using CLSM to observe microspheres stained by DAPI.

### 2.4 Detection of cellular uptake of nanomedicines *in vitro* and anti-inflammatory properties

The experimental groups included FITC-tagged PM NPs and SPM NPs. In this experiment, inflammatory HUVEC cells and Caco-2 cells were cultured in confocal microscopy dishes, with three dishes prepared for each group. FITC-SPM NPs (0.5 mg/mL) or FITC-PM NPs (0.5 mg/mL) were added to the respective dishes. Cells were then fixed with paraformaldehyde, washed three times with serum-free medium to remove excess nanoparticles, digested with trypsin, and resuspended in PBS. Flow cytometry was subsequently employed to measure and compare the fluorescence intensities of FITC-labeled nanoparticle uptake between the two groups. Statistical analysis was performed to determine whether sialic acid modification affected nanoparticle endocytosis. Confocal laser scanning microscopy (CLSM) was used to examine cellular fluorescence, assess differences in fluorescence intensity between groups, and quantify results using Image J. All experiments were conducted in triplicate. To assess the efficacy of SPM/AlgL in inhibiting the vascularization of inflammatory HUVEC cells, five distinct experimental groups were established. A 96-well plate was pre-cooled, and 50 μL of matrigel matrix gel was added to each well. Three wells were prepared for each group. The plates were incubated until the matrix gel solidified. A suspension of factor-stimulated inflammatory HUVEC cells (100 μL per well) was then dispensed into the wells. After a 4 h incubation, CLSM was used to observe and capture images of HUVEC cell vascularization. Comparative analysis was conducted to evaluate angiogenesis inhibition across the groups.

### 2.5 Establishment of rat RA animal model

Female Sprague‒Dawley (SD) rats (180‒200 g) were provided by Zhejiang Weitong Lihua Experimental Animal Technology Co., Ltd. (Jiaxing, China). The animals had free access to food and water and were housed in a ventilated room with a 12 h light/dark cycle. The room temperature was maintained at 20‒24 °C, and the humidity was kept at 40%–60%. Animals were fasted for 12 h before the experiment but had free access to water. All experimental procedures were executed according to the protocols approved by the Ethics Committee of Ruijin Hospital, affiliated with Shanghai Jiaotong University School of Medicine and strictly adhered to the guidelines of the Experimental Animal Ethics Committee.

A group of 4-week-old female SD rats received an intradermal injection of 150 μL of Freund's Complete Adjuvant (CFA) into the right hind paw. After 7 days of immunization, a successful animal model of rheumatoid arthritis was established and evaluated using a standardized scoring system.

### 2.6 Test system intestinal targeting capacity

The experiment comprised three groups: FITC, FITC-SPM NPs, and FITC-SPM/AlgL. Each group of rats received a 0.5 mg/mL microsphere suspension *via* oral gavage. At specific time intervals post-administration, the major organs and intestinal tissues of the rats were collected. These samples were then analyzed using IVIS to evaluate the adhesion and retention of the microsphere system in the intestinal tract and to generate imaging records.

### 2.7 Detection of the lymph node accumulation of SPM NPs

Intestinal lymph nodes were extracted from rats after a 12 h oral administration of SPM NPs and SPM/AlgL (both labeled with FITC). Frozen sections were prepared from these lymph nodes and analyzed using confocal laser scanning microscopy (CLSM) to measure fluorescence intensity. The analysis aimed to compare fluorescence intensities and assess the accumulation of nanomedicine in the lymph nodes.

### 2.8 Detecting the therapeutic effect of SPM/AlgL

Female AIA rats were randomly assigned to different treatment groups. Each group received intragastric administration of medicines every other day for 21 days. Specifically, each rat was administered 100 μg of melittin, SPM NPs, or SPM/AlgL microspheres containing melittin at a dose of 100 μg, *via* oral gavage every other day. Throughout the experimental period, the rats’ feet were photographed and evaluated, and their body weight was measured every 7 days. On Day 28, samples of the primary organs and feet were collected for histological staining with hematoxylin and eosin (H&E). Additionally, foot samples were examined using micro-CT imaging to assess the impact of orally administered anti-inflammatory substances. The efficacy of these substances was further analyzed through a comprehensive evaluation.

### 2.9 Detection of Th cells in the mesenteric lymph node and spleen of AIA rats

Mesenteric lymph nodes and spleens were harvested from each group of rats and placed into a cell strainer. The tissues were then minced and rinsed with RPMI 1640 medium. The resulting single-cell suspension was transferred to 15 mL centrifuge tubes and centrifuged at 500 × *g* for 5 min to pellet the cells and remove the supernatant. A total of 10^7^ cells were stimulated with lonomycin and golgistop for 4 h. The cells were then collected and placed into 1.5 mL centrifuge tubes. The cells were first blocked with a blocking antibody solution and washed. A fluorescent primary antibody specific to the surface proteins of interest was added to label the target proteins. After staining, the cells were washed three times with RPMI 1640 medium. The cells were then fixed with BD Cytofix/Cytoperm fixative to immobilize intracellular components. Following fixation, a fluorescent primary antibody was added to label the intracellular proteins of interest. The cells were washed again and resuspended in 100 μL of PBS. Flow cytometry was used to quantify the proportions of different Th cell subsets, and the data were analyzed statistically.

### 2.10 Enzyme linked immunosorbent assay and qRT-PCR for gene expression analysis

Fresh serum was collected from each group of rats and analyzed using ELISA to quantify the levels of inflammatory proteins IL-1*β*, IL-6, IL-17A, and IL-10. Serum samples were introduced into reaction wells and incubated at 37 °C for 1 h, followed by a washing step. Blank control wells were also prepared. Next, 0.1 mL of freshly diluted ELISA antibody was added to each reaction well and incubated at 37 °C for 30 min. After another washing step, 0.1 mL of freshly prepared TMB substrate solution was added to each well and allowed to react at 37 °C for 10–30 min. The reaction was terminated by adding 0.05 mL of 2 mol/L sulfuric acid to each well.

### 2.11 Statistical analysis

The experiment was repeated at least three times, and all data are expressed as mean ± standard deviation (SD). Statistical analysis was evaluated using a one-way ANOVA with Tukey's multiple comparison test by GraphPad Software to further evaluate the differences between groups. *P* values less than 0.05 were considered statistically significant (∗*P* < 0.05, ∗∗*P* < 0.01, ∗∗∗*P* < 0.005, ∗∗∗∗*P* < 0.001). “ns” was not significant.

## 3 Results

### 3.1 Synthesis and characterization of SPM/AlgL micro-nano system

**Figure 1:**
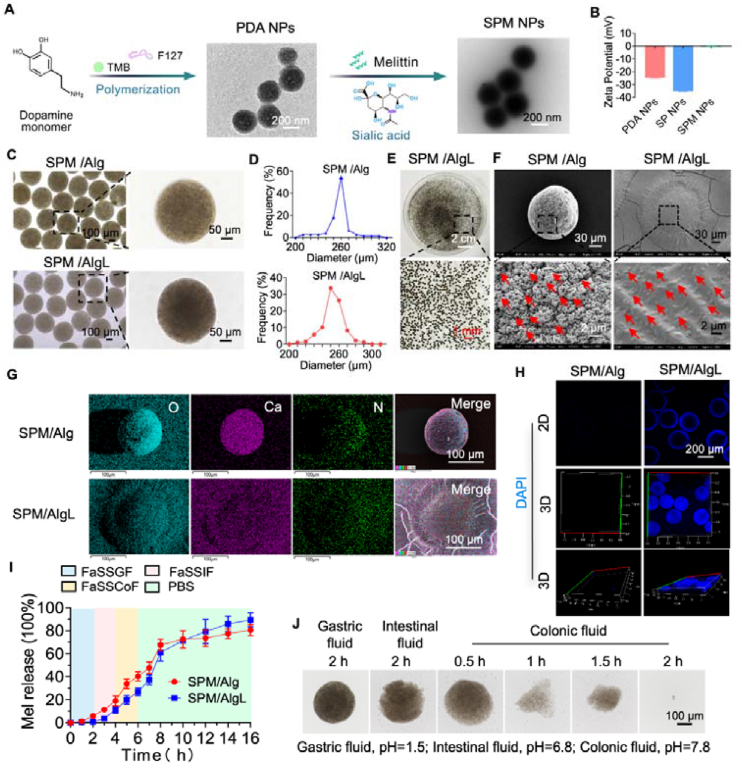
Preparation and characterization of the oral SPM/AlgL. (A) Preparation and TEM images of PDA NPs and SPM NPs. Scale bar = 200 nm; (B) Zeta potential data of the nanoparticles; (C) Optical microscope images of SPM/Alg and SPM/AlgL microspheres. Scale bar = 100 μm and 50 μm; (D) Statistical size distributions of SPM/Alg and SPM/AlgL microspheres obtained from microscopic brigh*t-*field observations; (E) Optical photographs of SPM/AlgL microspheres. Scale bars = 2 cm and 1 mm; (F) SEM images of SPM/Alg and SPM/AlgL microspheres. Scale bar = 30 and 2 μm; (G) EDS images of SPM/Alg and SPM/AlgL microspheres. Scale bar = 100 μm; (H) Confocal laser scanning microscope images of SPM/Alg and SPM/AlgL microspheres. Scale bar = 200 μm; (I) Kinetics of melittin release from SPM/AlgL in Fasted State Simulated Gastric Fluid (FaSSGF), Fasted State Simulated Intestinal Fluid (FaSSIF), Fasted State Simulated Colonic Fluid (FaSSCoF), and PBS buffer (pH7.4); (J) Microscopic brigh*t-*field observations of the morphological changes of SPM/AlgL microspheres after incubation in gastric fluid for 2 h, intestinal fluid for 2 h, and colonic fluid for 2 h. Scale bar = 100 μm.

Despite its excellent therapeutic efficacy in treating rheumatoid arthritis (RA), the clinical application of melittin is severely limited by its lack of specific targeting, potential risk of hemolysis, and propensity to cause tissue damage. To address these limitations, we synthesized mesoporous polydopamine nanoparticles (PDA NPs) as a backbone material with a specific surface structure. These nanoparticles exhibited an average size of 250 nm and a negatively charged surface (Zeta potential of approximately −27 mV) (Fig. 1A and B). To enhance targeting to inflammatory sites, the surface of PDA NPs was modified with sialic acid (SA), resulting in SP NPs. This modification decreased the electronegativity of the nanoparticles to −37 mV, confirming the successful incorporation of SA (Fig. 1B). Melittin was then effectively encapsulated within these SP NPs through robust physical adhesion and potent electronegativity, yielding SPM NPs. This encapsulation not only improved targeting to inflammatory vessels but also ensured the secure containment of the highly cationic melittin within the nanoparticle's mesoporous structure, with minimal alteration in nanoparticle size (Fig. 1A and B). The shift in surface charge towards neutrality (∼0 mV) confirmed the effective encapsulation of melittin, thereby enhancing its specific distribution and safety *in vivo* (Fig. 1B).

The inherent acid sensitivity of SPM NPs limits their effectiveness for oral administration, as melittin is prone to degradation in the gastrointestinal tract. To address this challenge, we developed a calcium alginate hydrogel microsphere system using a gas-shear microfluidic technique. The resulting SPM/Alg microspheres had an average size of approximately 260 μm (Fig. 1C and D). These microspheres, characterized by their gel-like structure and high water content, provided an optimal environment for nanomedicine storage. They are contracted in gastric fluid and dissolved in intestinal fluid, making them suitable for oral delivery. However, the porous structure of calcium alginate microspheres allowed gastric fluid permeation, leading to the inactivation of encapsulated melittin. To further improve the stability of these microspheres in acidic environments, we drew inspiration from the self-coating biofilm of *Lactobacillus reuteri*. We encapsulated a probiotic biofilm onto the surface of SPM/Alg microspheres, resulting in the formation of SPM/AlgL micro-nano systems with a size of about 250 μm and good dispersibility in water (Fig. 1E). Bacterial biofilms are naturally resistant and impermeable to gastric acid due to their dense physical structure and polysaccharide and nucleic acid-rich extracellular polymeric substance (EPS). This effectively retained the biological activity of melittin. Scanning electron microscopy (SEM) analysis revealed that the biofilm effectively covered the surface of the SPM/AlgL microspheres, with *Lactobacillus reuteri* evenly distributed within the biofilm (Fig. 1F). Energy-dispersive spectroscopy (EDS) confirmed the biofilm encapsulation, as the signals of Ca and N elements were masked on the surface of the SPM/AlgL microspheres, likely due to the presence of the biofilm (Fig. 1G and Supporting Information Fig. S1). DAPI staining and confocal microscopy further verified the stability and uniformity of the biofilm shell, demonstrating the high biological activity of *Lactobacillus reuteri* and its strong antacid and pro-intestinal adhesive properties (Fig. 1H).

The release behavior of melittin from SPM/AlgL microspheres was evaluated in simulated gastrointestinal fluids. Both SPM/Alg and SPM/AlgL microspheres exhibited slow melittin release in simulated gastric fluid. However, the release rate accelerated after 2 h of incubation in simulated intestinal fluid, with rapid release observed in PBS at pH7.4. These phenomena may be attributed to the ionic crosslinking-based gelation mechanism and the pH sensitivity of alginate hydrogel. Specifically, the release rate of melittin appears to depend on structural changes in alginate under different pH conditions. Alginate contains carboxyl groups (―COOH) with a low dissociation constant (p*K*a: 3.5). In the strongly acidic environment of gastric fluid (pH1.5), these carboxyl groups become protonated, causing the calcium alginate hydrogel spheres to shrink due to the loss of negative charge. Consequently, melittin release is significantly hindered. When the pH exceeds 3.5, the carboxyl groups ionize, and the alginate molecules become highly charged. This increased negative charge enhances electrostatic repulsion within the alginate chains, leading to the expansion and swelling of the hydrogel microspheres, with the most significant effect observed around pH7.4. Notably, SPM/AlgL microspheres, coated with a biofilm, demonstrated a reduced rate of melittin release in gastric fluid compared to SPM/Alg microspheres, indicating enhanced protection against gastric degradation. The accelerated release rate in intestinal fluid is likely due to the responsive disintegration of the hydrogel microspheres. Cumulative melittin release was higher in the SPM/AlgL group than in the SPM/Alg group after 10 h, probably because melittin in the SPM/Alg group was more extensively degraded in the stomach (Fig. 1I). The dual protection provided by the biofilm and calcium alginate enabled SPM/AlgL microspheres to resist gastric erosion while ensuring targeted drug release in the intestinal tract. Microscopic analysis further confirmed their stability in artificial gastric fluid (pH1.5) and subsequent dissolution in artificial small intestinal fluid (pH6.8). Upon incubation in artificial colon fluid (pH7.8), the microspheres rapidly disintegrated, releasing black particles, and were fully dissolved within 2 h (Fig. 1J). These results demonstrate that SPM/AlgL microspheres can effectively resist gastric acid while ensuring targeted drug release in the colon, thereby providing a robust foundation for the oral delivery of melittin.

### 3.2 Exploration of cellular uptake of SPM and inflammatory targeting mechanism

**Figure 2:**
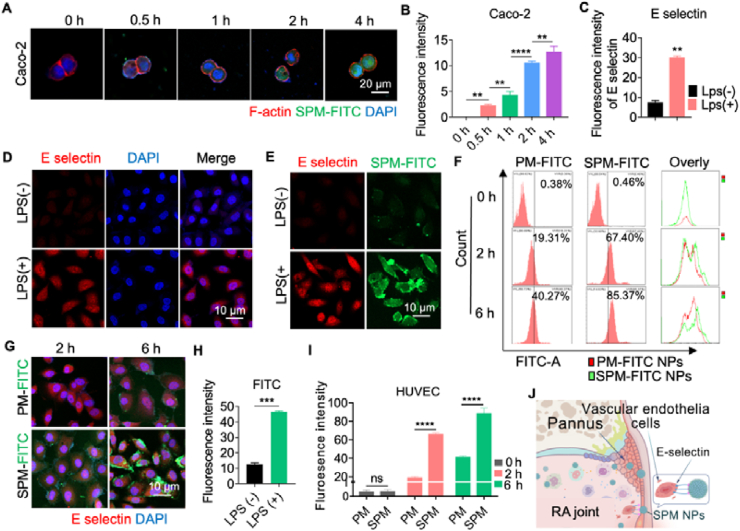
The uptake and the mechanism of SPM NPs *in vitro*. (A) The LCSM images and (B) the fluorescence intensity analysis of Caco-2 cells incubated with SPM-FITC for 0, 0.5, 1, 2, and 4 h. Scale bar = 20 μm; (C) The fluorescence intensity analysis of the E selectin expression and (D) the LCSM images of SPM-FITC NPs co-incubated HUVEC cells with or without LPS stimulation. Scale bar = 10 μm; (E) The LCSM images and (H) the fluorescence intensity analysis of SPM-FITC NPs co-incubated HUVEC cells with or without LPS stimulation. Scale bar = 10 μm; (F) The flow cytometry data and (I) the fluorescence intensity analysis of HUVEC cells incubated with FITC labeled PM NPs (PM-FITC) or SPM-FITC NPs after LPS stimulation at 0, 2, and 6 h, respectively; (G) The LCSM images of the PM-FITC or SPM-FITC NPs co-incubated HUVEC cells after LPS stimulation at 2 and 6 h. Scale bar = 10 μm; In RA joints, SPM NPs bind to E-selectin on inflammatory HUVECs within the synovial pannus, promoting their retention in the joint. (J) The encapsulated melittin is subsequently released, inhibiting the proliferation of inflammatory blood vessels. Data are presented as mean ± SD (*n* = 3). Significance levels: ∗∗*P* < 0.01, ∗∗∗*P* < 0.001, ∗∗∗∗*P* < 0.0001 were determined by *t-*test or one-way ANOVA with Tukey's multiple comparison test (*n* = 3). “ns” indicates not significant. LCSM, laser confocal scanning microscope; SPM-FITC NPs, FITC labeled SPM NPs; PM-FITC NPs, FITC labeled PM NPs.

To achieve therapeutic efficacy in rheumatoid arthritis (RA), SPM NPs must first be internalized by intestinal epithelial cells, then accumulate and migrate through systemic circulation to reach inflamed regions within the body. To evaluate this process, we monitored the uptake of FITC-labeled SPM NPs by Caco-2 cells over time using confocal laser scanning microscopy (CLSM). SPM NPs at a concentration of 10 μg/mL showed no obvious toxicity to Caco-2 cells, so we selected this concentration for our experiments (Supporting Information Fig. S2). The results demonstrated a time-dependent increase in nanoparticle uptake, confirming the effective internalization of SPM NPs by intestinal cells and establishing their potential for transport into the bloodstream (Fig. 2A and B).

Patients with rheumatoid arthritis (RA) often exhibit angiogenesis and vascular dysfunction, with endothelial cells playing a key role in these processes. Although human umbilical vein endothelial cells (HUVECs) are not directly derived from RA lesions, they are widely used to study the inflammatory response associated with endothelial cells. Under inflammatory conditions, HUVECs significantly enhance their role in mediating inflammation by upregulating adhesion molecules, such as E-selectin. Therefore, we investigated the interaction of SPM NPs with HUVECs under inflammatory conditions. SPM NPs at a concentration of 10 μg/mL showed no obvious toxicity to HUVECs as well, so this concentration was chosen for the experiments (Supporting Information Fig. S3). LPS stimulation significantly upregulated E-selectin expression on HUVECs compared to unstimulated controls (Fig. 2C and D), supporting the hypothesis that inflammation enhances sialic acid-mediated endocytosis of SPM NPs by HUVECs. To further validate this hypothesis, we conducted a comparative study of SPM NP uptake in LPS-stimulated (LPS+) *versus* unstimulated (LPS−) HUVECs. Confocal laser scanning microscopy (CLSM) and fluorescence intensity measurements revealed a marked increase in SPM NP uptake by LPS + HUVECs compared to LPS- HUVECs (Fig. 2E and H). This suggests that inflammation significantly enhances the internalization of peptide nanomedicines by endothelial cells. To explore the dynamics of this increased uptake, we performed co-culture experiments with LPS + HUVECs using both SP NPs and SPM NPs, followed by CLSM and flow cytometry analysis at 0, 2, and 6 h. The results demonstrated a time-dependent increase in nanoparticle uptake, with SPM NPs showing significantly higher uptake than SP NPs. Specifically, SPM NPs exhibited more than double the uptake compared to SP NPs at all time points (Fig. 2F, G and I, Supporting Information Fig. S4). This indicates that the enhanced uptake of SPM NPs by inflamed HUVECs is likely driven by the strong interaction between sialic acid on the nanoparticle surface and upregulated E-selectin on HUVECs during inflammation. Our study demonstrates that SPM NPs are effectively absorbed by intestinal epithelial cells and exhibit enhanced targeting and uptake in inflamed endothelial cells due to the interaction between sialic acid and E-selectin. This dual-targeting mechanism highlights the potential of SPM NPs as a promising therapeutic strategy for RA, capable of efficiently delivering nanomedicine to inflamed sites while minimizing off-target effects (Fig. 2J).

### 3.3 Evaluation of the targeting and anti-inflammatory effects of SPM/AlgL *in vivo*

**Figure 3:**
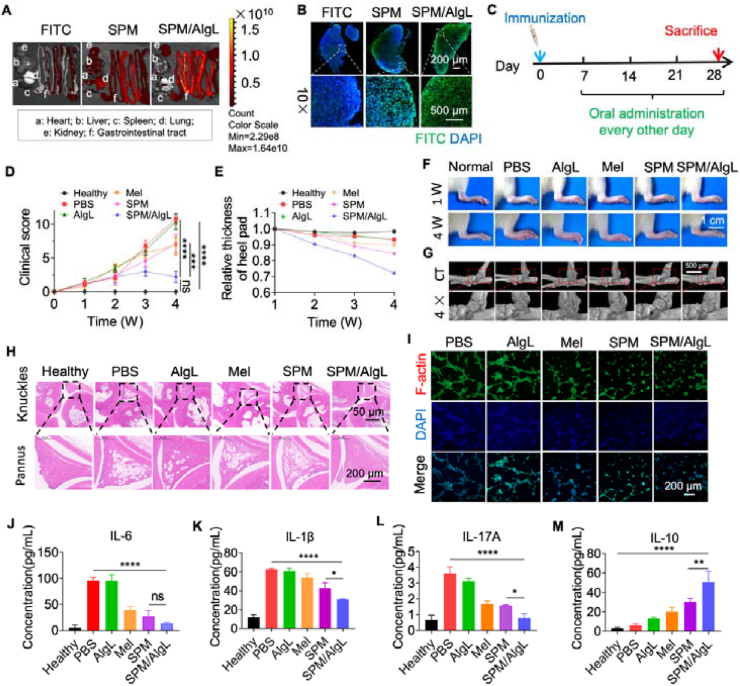
Evaluation of anti-rheumatoid arthritis efficacy *in vivo*. (A) Fluorescence imaging of AIA rat intestinal tissue demonstrating the adhesion ability of SPM/AlgL. (B) Fluorescence images of frozen sections of rat intestinal lymph nodes. Scale bar = 200 and 500 μm. Experimental Outline: The AIA model was established by immunizing SD rats. Arthritis fully developed seven days post*-*immunization. (C) AIA rats received various oral treatments and were evaluated throughout the 28 days' treatment period. Each group consisted of six rats (*n* = 3). (D) Average clinical scores for RA at predetermined time points in each group. (E) Changes in paw thickness for each rat in respective groups up to four weeks pos*t-*immunization. (F) Representative photos of hind paws in different treatment groups at 1 and 4 weeks post*-*immunization. Scale bar = 1 cm. (G) Micro-CT images of rat posterior foot ankle joints after different treatments. Scale bar = 500 μm. (H) H&E staining of rat foot knuckles. Scale bar = 50 and 200 μm. (I) Confocal laser scanning microscopy (CLSM) images of HUVEC cells incubated with PBS, AlgL, Mel, SPM NPs, and SPM/AlgL for 4 h. Scale bar = 200 μm. (J‒M) Concentrations of IL-6, IL-1*β*, IL-17A, and IL-10 in blood serum were measured using ELISA assays. Data are presented as mean ± SD (*n* = 3). Significance levels: ∗∗*P* < 0.01, ∗∗∗*P* < 0.001, ∗∗∗∗*P* < 0.0001 were determined by *t-*test or one-way ANOVA with Tukey's multiple comparison test (*n* = 3). “ns” indicates not significant. AIA, Adjuvan*t-*induced arthritis.

Before evaluating the therapeutic potential of the SPM/AlgL micro-nano system for rheumatoid arthritis (RA), we assessed its intestinal targeting capabilities and subsequent accumulation in lymph nodes. In the AIA rat model, orally administered FITC-SPM/AlgL exhibited significantly higher retention in the intestinal tract compared to free FITC and FITC-SPM NPs, as observed *via in vivo* imaging (Fig. 3A). This suggests that the biofilm-coated calcium alginate shell effectively enhances the intestinal retention of melittin nanomedicine, likely facilitating its absorption and transit in the intestine. Further analysis using DAPI staining on mesenteric lymph nodes confirmed enhanced accumulation of FITC-SPM/AlgL in these nodes compared to other groups (Fig. 3B). This indicates that the biofilm-coated microspheres not only protect the nanomedicine during transit but also enhance its delivery to the mesenteric lymph nodes. By targeting the intestinal immune system, this system potentially amplifies the therapeutic effects of melittin.

The therapeutic efficacy of the SPM/AlgL system was evaluated in a rat model of rheumatoid arthritis (RA). Female SD rats were divided into six groups and subjected to AIA induction. The rats were treated every other day for 21 days with either 100 μg of melittin, SPM NPs, or SPM/AlgL microspheres containing melittin (100 μg) *via* oral gavage (Fig. 3C). To assess the effectiveness of SPM/AlgL treatment, we recorded photographs of the plantar and ankle regions of rats at various time points. Over time, the SPM/AlgL group exhibited a substantial reduction in plantar and ankle swelling compared to the AIA group, outperforming other treatment groups. Additionally, RA-induced inhibition of weight gain was significantly alleviated in the SPM/AlgL-treated group compared to healthy rats (Supporting Information Fig. S5). The SPM/AlgL-treated group demonstrated significant reductions in plantar thickness and clinical scores compared to the PBS group. The reduction in clinical scores was more pronounced in the SPM/AlgL group than in the SPM group. Changes in plantar plate thickness were recorded at different time points, and statistical analysis showed that the plantar thickness of AIA rats in the SPM/AlgL group improved significantly by the end of treatment, indicating enhanced therapeutic efficacy (Fig. 3D–F, Supporting Information Fig. S6). Micro-CT analysis further revealed reduced bone erosion and joint stenosis in the ankle joints of the SPM/AlgL group (Fig. 3G), highlighting the system's efficacy in mitigating RA-induced joint damage. The inhibition of excessive angiogenesis, a hallmark of RA, was confirmed through H&E staining of rat joint sections. The SPM/AlgL group exhibited reduced synovial membrane proliferation and vascular opacities compared to the PBS group, with more effective suppression of angiogenesis than in the AlgL, Mel, and SPM-treated groups (Fig. 3H). To further elucidate this effect, *in vitro* cellular experiments were conducted. Consistent with the *in vitro* findings in LPS stimulated HUVECs, SPM NPs and SPM/AlgL significantly inhibited angiogenesis, underscoring the potent anti-inflammatory properties of melittin nanomedicine (Fig. 3I).

Lastly, serum samples were collected from each group of rats after treatment. ELISA results revealed significant reductions in pro-inflammatory cytokines (IL-6, IL-1*β*, IL-17A) and a notable increase in the anti-inflammatory cytokine IL-10 in the SPM/AlgL-treated group compared to PBS controls. The SPM/AlgL group demonstrated superior efficacy compared to other treatment groups. Specifically, SPM/AlgL exhibited a more pronounced inhibitory effect on IL-1*β* and IL-17A and a stronger stimulatory effect on IL-10 secretion compared to SPM. Although SPM/AlgL showed a slightly lower inhibitory effect on IL-6 than SPM, both treatments significantly reduced IL-6 levels compared to the PBS group (Fig. 3J‒M). These findings confirm that SPM/AlgL not only mitigates local inflammation in RA-affected tissues but also shifts the systemic immune response towards an anti-inflammatory state. In summary, the SPM/AlgL micro-nano system effectively targets the intestinal tract and mesenteric lymph nodes, enhancing the localized delivery of melittin nanomedicine. This targeted approach significantly suppresses RA-associated inflammation and joint damage, highlighting its potential as a novel oral therapeutic strategy.

### 3.4 Immunomodulation of the mesenteric lymphatic system by SPM/AlgL treatment

**Figure 4:**
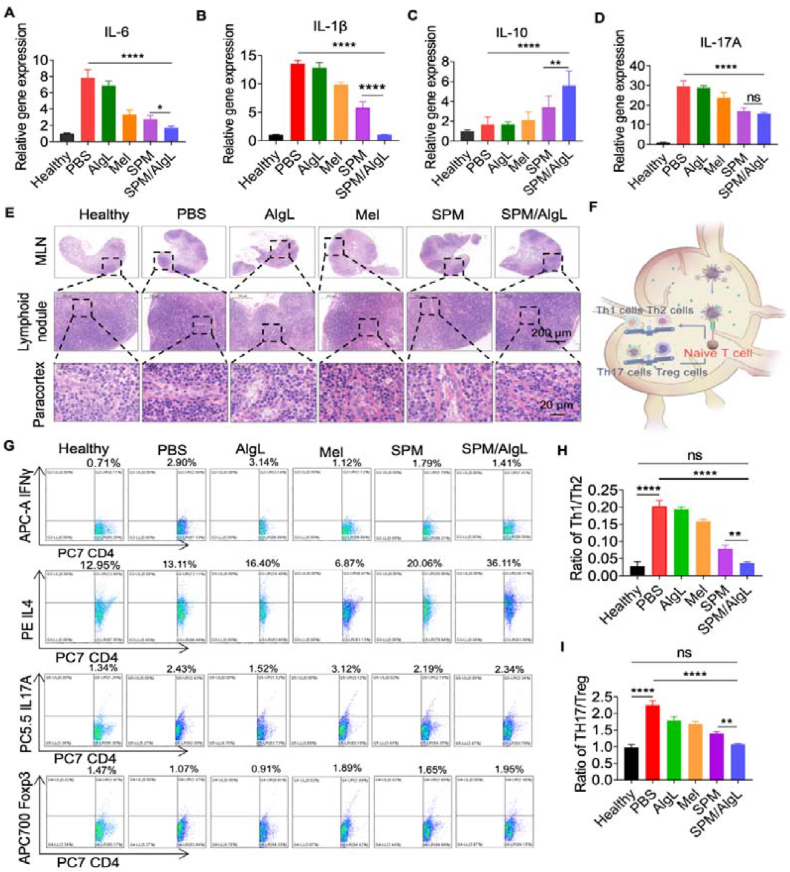
Immunomodulation of the mesenteric lymphatic system. (A‒D) The mRNA expression levels: the expression levels of IL-10, IL-1*β*, IL-6, and IL-17A in mesenteric lymph nodes. (E) Histological Analysis: H&E staining of rat intestinal lymph nodes. Scale bar = 200 and 20 μm. (F) Schematic diagram: Illustration of immune changes at the intestinal site. (G) Immune cell extraction: CD4+Th1, CD4+Th2, CD4+Th17, and CD4+Treg cells were extracted from the mesenteric lymph nodes of rats from different groups (*n* = 3). (H, I) Flow cytometry analysis: statistical results of Th17/Treg ratios. Data are presented as mean ± SD (*n* = 3). Significance levels: ∗*P* < 0.05, ∗∗*P* < 0.01, ∗∗∗*P* < 0.001 were determined by *t-*test or one-way ANOVA with Tukey's multiple comparison test (*n* = 3).

To investigate the immune regulation and inflammatory suppression in the intestinal region of RA rats following oral administration of the SPM/AlgL micro-nano system, we performed qRT-PCR analysis on colon tissues from each treatment group. The results indicated significant reductions in the expression levels of pro-inflammatory cytokines IL-1*β*, IL-6, and IL-17A in the SPM/AlgL group compared to the PBS group. Compared with the SPM group, SPM/AlgL more significantly inhibited the mRNA expression of IL-6 and IL-1*β*. Both treatments exhibited strong inhibitory effects on IL-17A mRNA expression, with no significant difference between them (Fig. 4A‒C). These cytokines are associated with Th cells, and their downregulation suggests a controlled inflammatory response. In contrast, the anti-inflammatory cytokine IL-10, which is associated with Treg cells, was significantly elevated in the SPM/AlgL group. SPM/AlgL had a stronger ability to promote IL-10 expression compared to the SPM group (Fig. 4D). This enhanced IL-10 expression in the intestinal tract indicates successful immunomodulation by SPM/AlgL treatment, leading to a notable decrease in intestinal inflammation. Further histological analysis *via* H&E staining of mesenteric lymph nodes revealed a marked increase in the number of immunoblasts with vacuolated cytoplasm within the internal paracortical area of lymph nodes in the AIA model. These immunoblasts likely reflect an acute inflammatory response. Notably, the SPM/AlgL-treated group exhibited a significantly lower number of immunoblasts compared to other groups, demonstrating the potent anti-inflammatory effects of the SPM/AlgL system (Fig. 4E).

These findings underscore the efficacy of the SPM/AlgL system in modulating the immune response within the intestinal lymphatic system a critical pathway in controlling systemic inflammation. The observed reduction in pro-inflammatory cytokine expression, coupled with the corresponding increase in anti-inflammatory markers, indicates that the SPM/AlgL micro-nano system effectively restores immune balance. This is achieved by enhancing Treg activity while suppressing pathogenic responses of Th cells. This dual modulation is essential for managing RA-related inflammation, both within the intestinal tract and systemically, as it likely prevents the cascade of immune responses that drive the progression of RA.

The immune response within the intestinal lymphatic system can significantly influence systemic immunity, primarily through lymphatic circulation. To explore the effects of oral SPM/AlgL treatment on peripheral immune balance, we analyzed the mesenteric lymph nodes of treated rats using flow cytometry. The results demonstrated a notable reduction in the Th1/Th2 and Th17/Treg cell ratios in the SPM/AlgL group compared to the PBS group. Compared with SPM, SPM/AlgL more significantly reshaped the balance of Th1/Th2 and Th17/Treg cells in the mesenteric lymph nodes. It also promoted the differentiation of Th0 cells towards Th2 and Treg cells and exhibited a stronger ability to induce immune tolerance (Fig. 4F‒I). These findings indicate that oral administration of SPM/AlgL effectively regulates peripheral immune balance in AIA rats, suggesting broader modulation of systemic immune responses.

### 3.5 Modulation of peripheral immune balance by oral SPM/AlgL treatment

**Figure 5:**
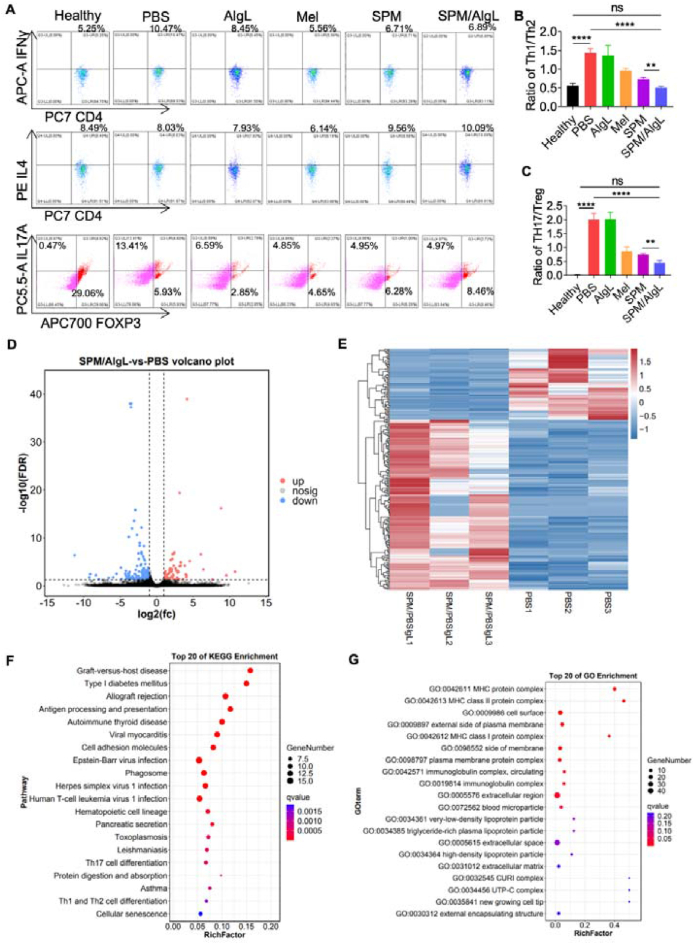
The spleen immune regulation *in vivo* of SPM/AlgL. (A) Immune cell extraction: CD4^+^Th1, CD4^+^Th2, CD4^+^Th17, and CD4^+^Treg cells were extracted from the spleens of rats from different groups (*n* = 3). (B) Flow cytometry analysis: statistical results of Th1/Th2 ratios. (C) Statistical results of Th17/Treg ratios. (D) Differential gene expression analysis: differential gene volcano map comparing the PBS and SPM/AlgL groups. (E) Differential gene heat map comparing the PBS and SPM/AlgL groups. (F) Pathway analysis: KEGG analysis of the PBS and SPM/AlgL groups. (G) GO analysis of the PBS and SPM/AlgL groups. Data are presented as mean ± SD (*n* = 3). Significance levels: ∗*P* < 0.05, ∗∗*P* < 0.01, ∗∗∗*P* < 0.001 were determined by *t-*test or one-way ANOVA with Tukey's multiple comparison test (*n* = 3).

Alterations in the immune response within the intestinal lymphatic system are likely to impact systemic immunity through lymphatic circulation. The presence of active lymphocytes in the mesenteric lymph nodes, as previously noted, suggests that peripheral immunity is also regulated concurrently. Following the completion of treatment, spleen tissues from rats in each group were collected and analyzed using flow cytometry. The results revealed that the ratios of Th1/Th2 and Th17/Treg cells in the SPM/AlgL group were significantly lower than those in the PBS group. Similarly, in the spleen, SPM/AlgL more effectively reshaped the balance of Th cell subsets compared to SPM. It promoted the differentiation of Th0 cells towards Th2 and Treg cells and exhibited a stronger capacity for immune regulation (Fig. 5A‒C). These findings indicate that oral administration of SPM/AlgL effectively modulates peripheral immune balance in AIA rats. Collectively, these results validate that oral SPM/AlgL treatment can influence Th cell differentiation, thereby effectively regulating immune equilibrium and managing inflammatory responses both in the intestinal tract and systemically.

To further investigate the immunomodulatory effects of the SPM/AlgL micro-nano system and its potential influence on immune signaling pathways, we performed RNA-seq analysis on spleen tissues collected from the rats. The data revealed significant alterations in immune-related signaling pathways, including those involved in antigen processing and presentation, in the AIA group compared to the SPM/AlgL group. The differential gene volcano map and heat map of the AIA and SPM/AlgL groups were highly concentrated in immune-related functions, suggesting that SPM/AlgL has a strong potential to control both immunity and inflammation (Fig. 5D and E). KEGG analysis highlighted changes in pathways associated with cellular senescence, endocytosis, and other critical immune processes. Notably, there were prominent modifications in genes related to immune presentation and signal transduction, which are critical in the development and progression of RA (Fig. 5F). Gene ontology (GO) enrichment analysis further highlighted the involvement of genes in pathways associated with antigen-antibody binding and the major histocompatibility complex (MHC) (Fig. 5G). Specifically, significant modifications were observed in the binding of MHC I and MHC II protein complexes to CD8^+^ and CD4^+^ T cells, respectively. These findings suggest that SPM/AlgL treatment may directly influence antigen presentation, potentially contributing to the observed therapeutic effects in RA. The SPM/AlgL micro-nano system can modulate peripheral immune balance by influencing Th cell differentiation and regulating key immune signaling pathways. This dual action not only addresses local inflammatory responses but also contributes to the overall management of systemic inflammation. These properties make SPM/AlgL a promising therapeutic strategy for RA. However, further studies are warranted to fully elucidate the mechanisms underlying these effects and to assess the long-term safety and efficacy of this approach.

### 3.6 Evaluation of SPM/AlgL microspheres on intestinal and systemic safety

**Figure 6:**
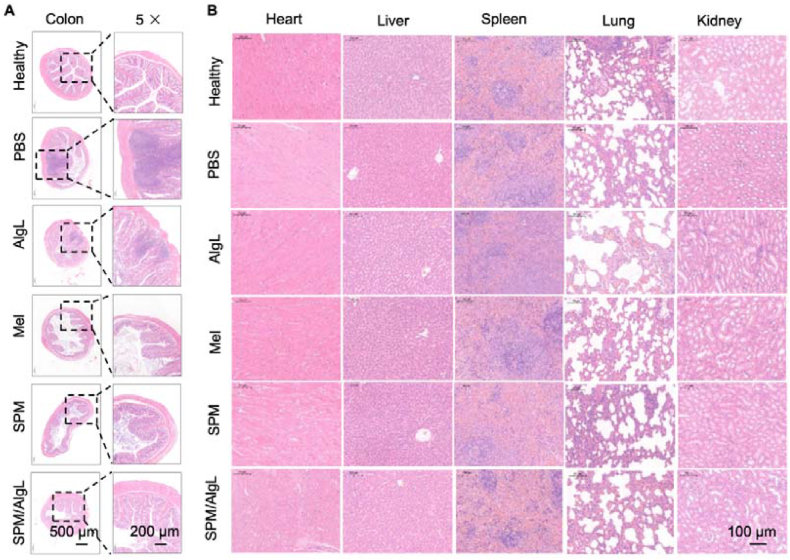
Biosafety evaluation after oral treatment. (A) The H&E images of the colon, Scale bar = 500 and 200 μm and (B) the major organs, Scale bar = 100 μm of rats from different groups, *n* = 3.

Rheumatoid arthritis (RA) is often accompanied by mild intestinal inflammation, which can be exacerbated by oral medications, leading to gastrointestinal damage and other adverse effects. To investigate the impact of oral SPM/AlgL treatment on intestinal tissues, colon sections from different groups of rats were analyzed using H&E staining. The results showed that compared to the Healthy, PBS, and AlgL groups exhibited mild intestinal inflammation, characterized by the presence of inflammatory cells in the colon region (Fig. 6A). These findings demonstrate that while melittin is effective in alleviating mild enteritis in RA rats, its direct oral administration poses a risk of slight damage to the intestinal mucosa. In contrast, the SPM/AlgL micro-nano system effectively mitigates these adverse effects, providing a safer and more protective approach to oral melittin administration.

To further assess the systemic safety of the developed micro-nano system, H&E staining was performed on sections of major organs (heart, liver, spleen, lungs, and kidneys) from rats after oral treatment with SPM/AlgL. The results revealed no noticeable damage to these organs across all treatment groups, including those receiving SPM/AlgL (Fig. 6B). This suggests that oral administration of melittin, at the doses used in this study, does not induce systemic toxicity. The micro-nano system exhibits high biocompatibility. The micro-nano encapsulation not only enhances the therapeutic efficacy of melittin by ensuring targeted release but also significantly reduces the risk of off-target effects and systemic toxicity.

## 4 Discussion

Oral delivery systems for rheumatoid arthritis (RA) therapy offer distinct advantages over injectable approaches, including improved patient compliance, reduced risk of hemolysis, and lower infection rates. However, the efficacy of oral delivery, particularly for biological drugs, is significantly hindered by the harsh pH conditions of the gastrointestinal tract and the abundance of digestive enzymes, which can degrade therapeutic agents before they reach their target sites. To address these challenges, we successfully developed a novel oral micro-nano system for the administration of melittin, inspired by the concept of “popping boba”. This system effectively targets therapeutic agents in the gut while preserving their bioactivity and minimizing systemic side effects. By utilizing calcium alginate hydrogel microspheres encapsulated with a *Lactobacillus reuteri* biofilm, we created a robust platform capable of protecting melittin potent but unstable therapeutic peptide from degradation in the gastric environment. The designed system facilitates the precise release of melittin-loaded nanoparticles in the alkaline intestinal environment, leading to selective accumulation of the drug in mesenteric lymph nodes. Our findings demonstrate that this targeted delivery approach significantly enhances the therapeutic efficacy of melittin by modulating the balance between Th cell subsets, particularly by increasing the ratio of Treg and Th2 cells in both mesenteric lymph nodes and the spleen. The consequent reduction in pro-inflammatory cytokine levels and improvement in immune homeostasis underscores the potential of this strategy not only for RA but also for broader applications in systemic autoimmune diseases.

## 5 Conclusions

Overall, this research introduces a new paradigm for achieving localized immunomodulation and oral peptide delivery through an innovative combination of biofilm protection and gas-shearing technology. Future work will focus on further optimizing this system for clinical translation and exploring its applicability to other challenging therapeutic targets. The novel approach outlined here holds promise for improving patient outcomes and advancing the treatment landscape of rheumatoid arthritis (RA) and other autoimmune conditions.

## Author contributions

Fangke Zhang, Weisheng Guo, Wenguo Cui, and Weiguo Hu conceived and designed the experiments; Fangke Zhang, Tao Ding, Jiancheng Zheng, and Xuefei Wang performed the experiments; Fangke Zhang, Tao Ding, Jiancheng Zheng, Xuefei Wang, Yawei Du analyzed the data; Weisheng Guo, Wenguo Cui and Weiguo Hu contributed reagents, materials and analysis tools; Fangke Zhang, Weisheng Guo, Nan Li, Zechuan Li wrote the paper. All of the authors have read and approved the final manuscript.

## Conflicts of interest

The authors have no conflicts of interest to declare.

## Appendix A Supplementary data

The following is the Supporting Information to this article.
