# Supplementary material for: Oral administration of probiotic colony-like micro-nano system for immunoregulation of rheumatoid arthritis
Source: Acta Pharm Sin B. 2025 Oct 31;16(1):444–57. doi: 10.1016/j.apsb.2025.10.038 (PMC12827885; doi:10.1016/j.apsb.2025.10.038)
Supplement: Multimedia component 1 [file mmc1.pdf]

Supporting information for

Original article

Oral administration of probiotic colony-like micro-nano system for immunoregulation of rheumatoid arthritis

Fangke Zhang[a,b,c,†] , Tao Ding[b†], Jiancheng Zheng[b†], Nan Li[a], Zechuan Li[a], Xuefei Wang[c], Yawei Du[b], Weiguo Hu[c,*], Wenguo Cui[b,*], Weisheng Guo[a,*]

[a]*Department of Minimally Invasive Interventional Radiology, The Second Affiliated Hospital, School of Biomedical Engineering Guangzhou Medical University, Guangzhou 510260, China*

[b]*Department of Orthopaedics, Shanghai Key Laboratory for Prevention and Treatment of Bone and Joint Diseases, Shanghai Institute of Traumatology and Orthopaedics, Ruijin Hospital, Shanghai Jiao Tong University School of Medicine, Shanghai 200025, China*

[c]*Department of Geriatrics, Medical Center on Aging, Ruijin Hospital, Shanghai Jiao Tong University, School of Medicine, Shanghai 200025, China*

Received 27 April 2025; received in revised form 20 June 2025; accepted 12 September 2025

*Corresponding authors.

E-mail addresses: guo_wei_sheng@gzhmu.edu.cn (Weisheng Guo), wgcui@sjtu.edu.cn (Wenguo Cui), wghu@rjh.com.cn (Weiguo Hu).

[†]These authors made equal contributions to this work.

Running title: Oral probiotic colony-like micro-nano system for immunoregulation of rheumatoid arthritis

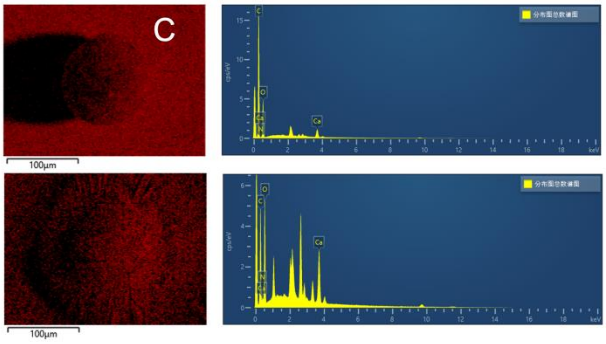

Figure S1. The EDS images about C element and analytical mapping of the SPM/Alg and the SPM/AlgL microspheres.

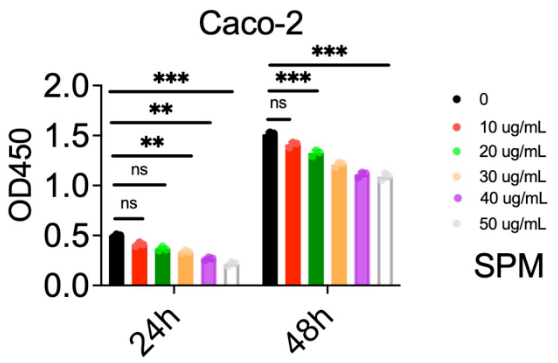

Figure S2. The viability of Caco-2 cells upon the addition of SPM NPs.

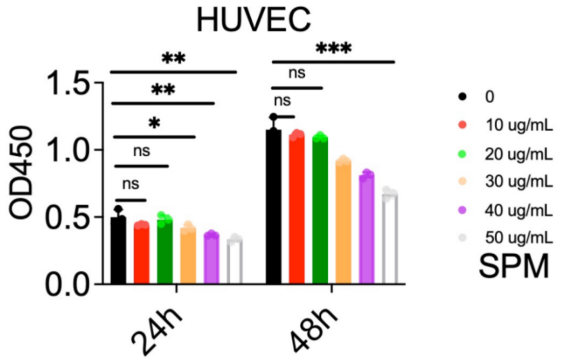

Figure S3. The viability of the HUVEC cells upon the addition of SPM NPs.

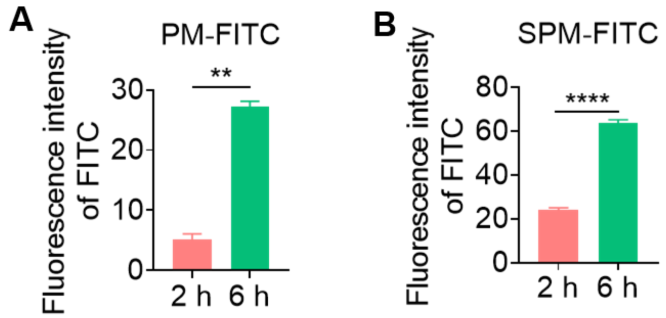

Figure S4. The fluorescence intensity analysis of HUVEC cells incubated with FITC labeled PM NPs (**A**) and SPM-FITC NPs (**B**) after LPS stimulation at 2 and 6 h, respectively.

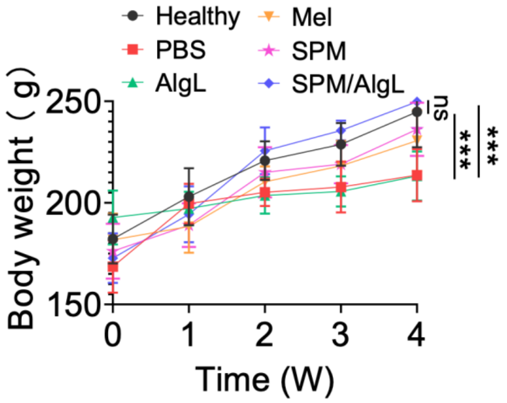

Figure S5. The body weight of RA rats in pre-determined time points in each group.

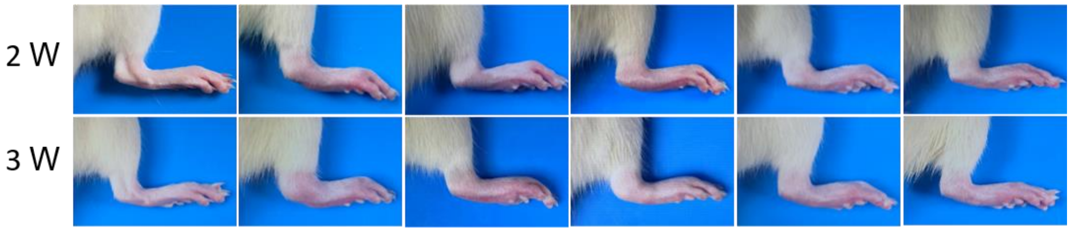

Figure S6. Representative photos of hind paw in different treatment groups 2 and 3 weeks after the first immunization.
